# Drink a Toast to Tap Water: Study Suggests Water Consumption Benefit Outweighs THM Hazard

**DOI:** 10.1289/ehp.115-a551a

**Published:** 2007-11

**Authors:** John Tibbetts

Many water treatment systems use chlorine to disinfect drinking water. However, chlorine reacts with dissolved organic matter in water to create trihalomethanes (THMs), which have been associated with excess risk of bladder cancer in people who drink chlorinated water. Now, in a large study conducted in Spain, an international team of scientists has examined the role of tap water and total fluid intake in bladder cancer risk, while also assessing the effect of exposure to THMs in water **[*EHP* 115:1569–1572; Michaud et al.]**. The results suggest that higher water consumption is associated with lower risk of bladder cancer, regardless of THM exposure.

Some studies have linked high consumption of fluids including tap water with a lower risk of bladder cancer, perhaps because urinating more frequently allows more flushing of the bladder. Other studies suggest that high tap water consumption could increase bladder cancer risk if chlorination by-products or other water contaminants such as arsenic are elevated in the water source. Adding to this complexity is that still other studies have shown a THM-related excess risk of bladder cancer in men but not women.

Between June 1998 and June 2001, researchers conducted a hospital-based case–control study of bladder cancer in multiple centers in Spain. Male and female bladder cancer patients aged 20–80 years were recruited from 18 participating hospitals. For the 397 bladder cancer cases available for this analysis, the team recruited 664 matched controls who had been admitted to the same hospitals around the same time for hernias, fractures, orthopedic problems, and other reasons.

Trained interviewers collected information during each subject’s hospitalization that included sociodemographic characteristics, family history of cancer, smoking history, occupational history, residential history, drinking water source at each residence, beverage consumption (including water), and medical history. The researchers used local government and water company data on annual average THM levels, water source history since 1920, and chlorination history to calculate average year-by-year THM exposure. These data were available for 78.5% of the total study person-years.

The researchers examined the association between total fluid and water consumption and bladder cancer risk, while also examining the interaction between water intake and THM exposure. The results suggest that drinking more water, even from chlorinated sources with high THM levels, is beneficial in reducing risk of bladder cancer. The authors found a 53% lower risk of bladder cancer in people who drank 1,400 mL or more water per day compared with those who drank less than 400 mL per day after adjusting for known and potential confounders. This inverse association held across all strata of smoking status and THM exposure, and for both men and women.

The study was strengthened by high response rates from cases and controls, detailed interview data on individual beverage consumption, detailed assessment of THM exposure, and detailed data on smoking, which is believed to be the greatest risk factor for bladder cancer.

## Figures and Tables

**Figure:**
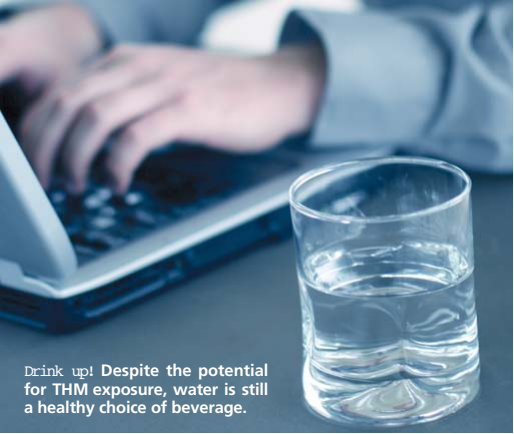
Drink up! **Despite the potential for THM exposure, water is still a healthy choice of beverage.**

